# DRSN4mCPred: accurately predicting sites of DNA N4-methylcytosine using deep residual shrinkage network for diagnosis and treatment of gastrointestinal cancer in the precision medicine era

**DOI:** 10.3389/fmed.2023.1187430

**Published:** 2023-05-04

**Authors:** Xia Yu, Jia Ren, Yani Cui, Rao Zeng, Haixia Long, Cuihua Ma

**Affiliations:** ^1^School of Information and Communication Engineering, Hainan University, Haikou, Hainan, China; ^2^School of Information Science and Technology, Hainan Normal University, Haikou, Hainan, China; ^3^Industrial Design School, Shandong University of ART and Design, Jinan, Shandong, China

**Keywords:** DNA N4-methylcytosine, site prediction, deep learning, deep residual shrinkage network, multi-scale channel attention

## Abstract

**Introduction:**

The DNA N4-methylcytosine (4mC) site levels of those suffering from digestive system cancers were higher, and the pathogenesis of digestive system cancers may also be related to the changes in DNA 4mC levels. Identifying DNA 4mC sites is a very important step in studying the analysis of biological function and cancer prediction. Extracting accurate features from DNA sequences is the key to establishing a prediction model of effective DNA 4mC sites. This study sought to develop a new predictive model, DRSN4mCPred, which aimed to improve the performance of the predicting DNA 4mC sites.

**Methods:**

The model adopted multi-scale channel attention to extract features and used attention feature fusion (AFF) to fuse features. In order to capture features information more accurately and effectively, this model utilized Deep Residual Shrinkage Network with Channel-Wise thresholds (DRSN-CW) to eliminate noise-related features and achieve a more precise feature representation, thereby, distinguishing the sites in DNA with 4mC and non-4mC. Additionally, the predictive model incorporated an inverted residual block, a Multi-scale Channel Attention Module (MS-CAM), a Bi-directional Long Short Term Memory Network (Bi-LSTM), AFF, and DRSN-CW.

**Results and Discussion:**

The results indicated the predictive model DRSN4mCPred had extremely good performance in predicting the DNA 4mC sites across different species. This paper will potentially provide support for the diagnosis and treatment of gastrointestinal cancer based on artificial intelligence in the precise medical era.

## 1. Introduction

Recently, artificial intelligence has achieved exciting achievements in many fields (1–3), which offers precision diagnosis and treatment services to human beings by combining various artificial intelligence techniques especially deep learning with medical theory (4). DNA 4mC is an epigenetic variation that may be associated with the occurrence of digestive system cancers. DNA methylation plays an essential role in defending against veracious repetitious element activity, gene silencing, genomic stability in the process of cell karyomitosis, etc. (5). In addition, the alteration of the DNA methylation pattern may lead to the occurrence of diseases, particularly cancers caused by environmental factors and aging (6, 7). The DNA 4mC sites defend host DNA against the degradation of restriction enzymes. Besides, it corrects the error of prokaryotic DNA replication, as well as regulates the DNA replication and generation cycle of prokaryotic organisms (8). Thus, the identification of DNA methylation is very important for studying the mechanisms of action in biology and medicine. Therefore, applying deep learning in artificial intelligence to detect DNA methylation sites can provide auxiliary functions for smart medicine.

However, traditional experimental techniques were used to detect the DNA methylation, which required higher costs (9). Moreover, due to the limitations of short-read sequencing, bisulfite sequencing could not describe DNA methylation in duplicate genomic regions (10, 11). For this reason, current research is increasingly concentrated on the development of intelligent methods to predict DNA methylation from DNA sequences directly, especially in machine learning. These DNA methylation identification methods were constructed as binary prediction tasks, and the machine learning models were trained to discriminate the actual methylation sites or not. In the past decades, many sequence-based models utilized a combination of conventional machine learning approaches and deep learning architectures to differentiate DNA 4mC sites. Chen (12) recently proposed an efficient prediction tool, iDNA4mC, that utilized the properties of nucleotide chemistry and frequency coding of DNA sequences to distinguish the 4mC sites. He (13) then proposed a second 4mC site prediction model, 4mCPred, which utilized novel feature encoding methods that combined the positional specificity of the trinucleotide trend and the pseudo-potentials of electron-ion interaction. Wei (14) proposed an iterative feature representation method for 4mC site prediction, which allowed the information features learned from several sequential models in the monitored iterative mode. Deep Torrent (15) was a deep learning-based predictive model; the model integrated an inception module, transfer learning, and attention module into the predictive model to improve the predictive performance. Jhabindra Khanal (16) proposed a 4mC-w2vec prediction tool that adopted distributed feature display method and a word embedding technique to discriminate the different species. Zeng (17) proposed the Deep4mcPred predictor, which automatically learned high-level features and captured specific characteristics to differentiate between 4mC sites or not. Wang (18) proposed a feature representation method that introduced the Pointwise Joint Mutual Information (PJMI) and bi-directional k-nucleotide Position-Specific Propensities (PSP), and the extraction of nucleotide position information was used to predict RNA methylation sites.

The i4mC-ROSE algorithm (19) was the first predictive model to predict 4mC sites of Rosaceae genomes and had been used to discriminate 4mC sites of *Fragaria vesca* (20) and *Rubia chinensis* (21) genomes. The 4mcDeep-CBI (22) deep learning framework proposed using a 3-convolution neural network (CNN) and Bi-LSTM (23) to obtain deep information and develop advanced features for discriminating 4mC sites in the DNA sequences of *Caenorhabditis elegans* (*C. elegans*). The DNC4mC-Deep (24) utilized several encoding techniques, which included 2Kmer (25), 3Kmer, binary encoding (26, 27), the chemical property and frequency of nucleotides (28) – along with a CNN and a grid search algorithm to perform 4mC site prediction across cross-species genomes.

The 4mCCNN (29) predictive model detected 4mC sites using a one-hot encoding matrix and CNN, but because the deep learning architecture of the model was small, it could not further expand its learning abilities (15). To improve predictive performance, the DNA4mC-LIP (30) model integrated six classical predictive models (12, 14, 30–32) and used a linear iterative strategy to explore and assign the best weights to each predictor. The comparison testing on independent test datasets revealed that the predictive performance was significantly enhanced. Additionally, the Hyb4mC (33) tool embedded sequences using the DNA2vec method and complemental networks, Hyb_Caps and Hyb_Conv, to get more accurate information than other methods based on the sequence features. Despite there being various predictive models for DNA 4mC sites, all the prediction performances were not very high, so the predictive performances need further improvement.

The primary objective of the paper was to enhance the performance of predicting 4mC sites. The DRSN4mCPred model used DRSN-CW to eliminate noise-related features and achieved a more accurate feature representation, allowing for better distinguishing of DNA 4mC sites or not. Additionally, inverted residual block, MS-CAM (34), Bi-LSTM (23), AFF (34), and DRSN-CW (35) were integrated into the prediction model. As a result, it was found that our predictor achieved superior performance in predicting the 4mC sites of different species. This research may support the diagnosis and treatment of digestive system cancers from an artificial intelligence perspective.

## 2. Materials and methods

### 2.1. Datasets

The research made use of the Hyb_2021 and Li_2020 datasets, both of which contained the species of *C. elegans*, *D. melanogaster*, *A. thaliana*, *E. coli*, *G. subterraneus*, and *G. pickeringii*. The Hyb_2021 dataset (33) was selected based on the technical methylation analysis (36, 37), ensuring that the IPD ratio for each position was evidently different from the expected background (the default value was modQV ≥30). In addition, these DNA sequences were 41 bp in length (12). As a comparison, we used the Li_2020 dataset (15) to prove that the proposed DRSN4mCPredmodel can predict 4mC sites across different species.

### 2.2. DRSN4mCPred

**Figure 1:**
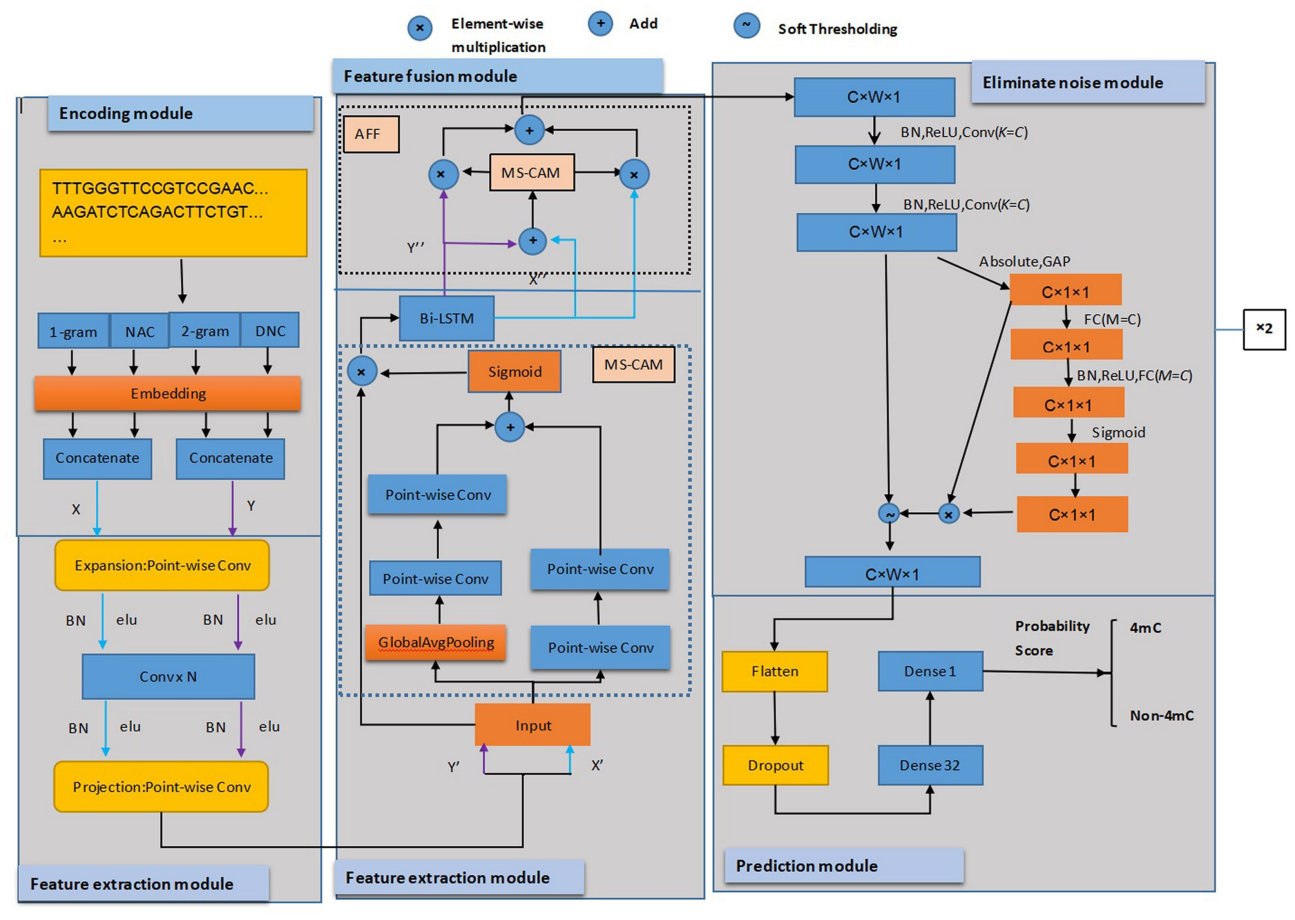
The framework of the proposed predictor. Conv was the 1 × 1 convolution layer; N represented the number of convolution layers. C, W, and one were the indicators of the number of channels, width, and height of the feature map, respectively. *K* is the number of convolution kernels in the convolution layer.

In this article, a novel prediction model was presented that integrated the feature extraction of multi-scale, fusion mechanism, and deep residual shrinkage network. The study’s innovation was to bring a deep residual shrinkage network into the prediction model to eliminate feature noise. As shown in Figure 1, our model consists of five modules: encoding, multi-scale feature extraction, feature fusion, noise elimination, and prediction.

The encoding module used four encoding schemes, 1-gram, 2-grams, NAC, and DNC (23), to represent DNA sequences, resulting in four feature matrices. The features of 1-gram and NAC encoding were combined, as well as the 2-grams and DNC encoding features, and then they were concatenated.

Next, in the extracting feature module, the matrices of the above two features were fed into the point-wise convolution layer and then supplied to the projection point-wise layer. We extracted more feature information using MS-CAM (34) network to combine the two matrices features of global and local before they were fused with a Bi-LSTM layer (23).

Third, in the fusion module, AFF (34) was used to concatenate the features that were extracted from the Bi-LSTM layer with addition operation, and the concatenated features were fed into the module of MS-CAM for calculating the weights of fusing two matrices features. We applied the fusion weights to the feature matrices through multiplication and combined results through addition operation, thus obtaining the features that aggregate global and local context features of four different coding methods.

Fourth, in the noise reduction module, we utilized DRSN-CW (35) to deal with the input features. The features were first processed by two 1D convolutions and then reduced using an absolute operation and Global Average Pooling (GAP). The resulting features were input into the two-layer Fully Connected (FC) network. There was more than one neuron in the two-layer FC network; the neurons’ count of FC matched the count of channels in the feature map of the input. Then we scaled the FC network’s output to ensure it fell within the range of zero and one, and soft thresholds were computed based on the scale parameters and features. The thresholds need to be within an appropriate range and have positive values to prevent all zero-output attributes. The soft thresholds were applied to features and then the original input features from MS-CAM were multiplied to obtain the final features that eliminated the noise-related features. In the eliminate noise module, the DRSN-CW was executed twice.

Finally, in the prediction module, the extracted features described above were processed by dropout layers to prevent overfitting during training. To better utilize the feature vectors of the preceding layers, we applied a flatten function to convert them into a single vector, and then we used the FC layer with 32 neurons. Ultimately, the sigmoid function was used in the FC layer, squeezing the values between zero and one for discriminating either DNA 4mC sites or not. During training, we used Adam Optimizer and implemented the loss function of binary cross-entropy. It predicted the score that determines the detection of 4mC sites in DNA sequences where a score higher than 0.5 indicated the 4mC sites existed, while a score lower than 0.5 indicated no 4mC sites existed. The model was coded using Keras 2.9.0. The model of DRSN4mCPred will be described in detail below.

#### 2.2.1. Encoding module

The DNA sequences are comprised of four nucleotides: Adenine (A), Thymine (T), Cytosine (C), and Guanine (G). S denoted the DNA sequence, $S=s_{1},s_{2},\cdots s_{i}\cdots ,s_{L}$, $s_{i}$ denoted the nucleotide at position i, and L represented the single DNA sequence’s length. The 4mC DNA sequences were encoded using the encoding methods, n-gram, NAC, and DNC.

##### 2.2.1.1. N-gram coding

N-grams were a set of all possible nucleobases’ subsequences (23). By setting the value of n to 1, we can generate 1-gram encoding features, and by setting it to 2, we can generate 2-gram encoding features. For 1-gram coding, the sequences were expressed as nucleotides ‘A’, ‘T’, ‘C’, and ‘G’; the nucleotides were mapped to numbers: A ~ 1, T ~ 2, C ~ 3, and G ~ 4. Thus, a DNA sequence can be mapped to a vector with a length of 41; the vectors were represented as $V_{i,1-\text{gram}}$.


(1) $$V_{i,1-\text{gram}}={\left[v_{1},v_{2},v_{3}\cdots v_{40},v_{41}\right]}_{i},v\in \left[1,2,3,4\right]$$

The 2-gram nucleotide sequences included ‘AA’, ‘AT’, ‘AG’, ‘TA’, ‘TC’, ‘TG’, ‘CA’, ‘CT’, ‘CG’, and ‘TT’. For example, the sequence ‘GAGGA … ACT’ can be encoded as ‘GA’, ‘AG’, ‘GG’, ‘GA’, …, ‘AC’, and ‘CT’. These encoded sequences were mapped with numbers from 1 to 16. With this dictionary, we can map any DNA sequence to a numerical vector of length 40; the vectors were denoted as $V_{i,2-\text{gram}}$.


(2) $$V_{i,2-\text{gram}}={\left[v_{1},v_{2},v_{3}\cdots v_{39},v_{40}\right]}_{i},v\in \left[1,2,3,\cdots 14,15,16\right]$$

##### 2.2.1.2. Nucleic acid composition (NAC) encoding

The NAC encoding method was used to calculate the frequency of nucleotide sequence for each nucleic acid type. The frequencies of the four nucleic types were represented as $f_{NAC}(t)$:


(3) $$f_{NAC}(t)=\frac{N(t)}{L},\;\;t\in \left\{A,T,C,G\right\}$$

The length of the DNA sequence was represented by L; $V_{i,NAC}$ represented the NAC encoding of the DNA sequence; the vector length of $V_{i,NAC}$ was four.


(4) $$V_{i,NAC}={\left[f_{NAC}(A),\;f_{NAC}(T),\;f_{NAC}(C),\;f_{NAC}(G)\right]}_{i}$$

##### 2.2.1.3. Di-nucleotide composition (DNC) encoding

The DNC encoding method was used to count the frequency of every two nucleotides in the DNA sequence, thereby representing the DNA sequence as 16 descriptors. The frequency was defined as $f_{DNC}$ of every two nucleotides in the DNA sequence.


(5) $$f_{DNC}\left(r,s\right)=\frac{N_{rs}}{L-1},r,s\in \left\{A,T,C,G\right\}$$

$N_{rs}$ represented the amount of di-nucleotide; the DNA sequence was denoted using a vector with a length of 16 recorded as $V_{i,DNC}$:


(6) $$V_{i,DNC}={\left[f_{DNC}\left(AA\right),f_{DNC}\left(AC\right),\cdots ,f_{DNC}\left(TC\right),f_{DNC}\left(TG\right)\right]}_{i}$$

Then, the vectors $V_{i,1-\text{gram}}$, $V_{i,NAC}$, $V_{i,2-\text{gram}}$, and $V_{i,DNC}$ were supplied to the embedding layer and transformed into learnable embedding vectors. A new vector $X_{i}$ was created by concatenating the embedding vectors of $\text{Embedding}\left(V_{i,1-\text{gram}}\right)$ and $\text{Embedding}\left(V_{i,\text{NAC}}\right)$. We performed an identical operation on the vectors of $V_{i,2-\text{gram}}$ and $V_{i,DNC}$, generating a new feature vector $Y_{i}$.


(7) $$X_{i}=\text{Con}\left(\text{Embedding}\left(V_{i,1-\text{gram}}\right),\text{Embedding}\left(V_{i,\text{NAC}}\right)\right)$$

(8) $$Y_{i}=\text{Con}\left(\text{Embedding}\left(V_{i,2-\text{gram}}\right),\text{Embedding}\left(V_{i,\text{DNC}}\right)\right)$$

In order to obtain accurate and effective features, $\;X_{i}$ and $Y_{i}$ were input to a subsequent module of extraction and fusion features.

#### 2.2.2. Multi-scale feature extraction and fusion

Multi-Scale Feature Extraction includes inverted residual block, MS-CAM, and Bi-LSTM module. In an inverted residual module (34), the input features were first passed through a 1×1 convolution layer, which was used to increase the number of channels. This was followed by an N-layer convolution layer to significantly reduce the number of network parameters and computation, which included a 1*1 convolution layer, 1D zero padding layer, batch normalization layer, and dropout layer. Finally, another 1×1 convolution layer was used to reduce the number of channels back to the original number.

##### 2.2.2.1. Multi-scale attention mechanism block

Multi-scale attention mechanism block (MS-CAM) (34) was capable of extracting both local features and global features and then combining the features with two feature matrices. To more accurately and effectively capture feature information, we used a Bi-LSTM (23) layer before the two feature matrices were fused. For a given feature X ∈ R^H × W × C^, the feature map had a dimension of H × W and was composed of C channels; the MS-CAM combined global and local features:


(9) $$M\left(X\right)=G\left(X\right)⊕L\left(X\right)$$

where G(X) represented global features and L(X) represented local features. The structure of MS-CAM is shown in Figure 1. G(X) ∈ R^C^ and L(X) ∈ R^C × H × W^(when H=W = 1, represents extracting global features information). The G(X) and L(X) were computed using the GAP and Batch Normalization (BN) as follows:


(10) $$L\left(X\right)=\text{BN}\left({\text{PWConv}}_{2}\left(\text{BN}\left({\text{PWConv}}_{1}\left(X\right)\right)\right)\right)$$

(11) $$G\left(X\right)=\text{BN}\left({\text{PWConv}}_{2}\left(\text{BN}\left({\text{PWConv}}_{1}\left(\text{GAP}\left(X\right)\right)\right)\right)\right)$$

(12) $$\text{GAP}\left(X\right)=\frac{1}{H\times W}\sum_{i=1}^{H}\sum_{j=1}^{W}X\left[:,i,j\right]$$

where the point-wise convolution (PWConv) was used for extracting local features, each separate filter size for PWConv_1_ and PWConv_2_ was (H × W × C)/r and H × W × C. Unlike the conventional MS-CAM, to preserve the feature information and prevent its destruction, we removed the non-linear activation function from the convolution layers. The expansion ratio of PWConv1 was r, and the expansion ratio of PWConv_2_ was 1/r. The refined features $X^{\prime }$ and $Y^{\prime }$ were expressed as:


(13) $$X^{\prime }=X⊗\sigma \left(M\left(X\right)\right)=X⊗\sigma \left(G\left(X\right)⊕L\left(X\right)\right)$$

(14) $$Y^{\prime }=Y⊗\sigma \left(M\left(Y\right)\right)=Y⊗\sigma \left(G\left(Y\right)⊕L\left(Y\right)\right)$$

where σ represented sigmoid function, ⊕ represented broadcasting addition, and ⊗ represented element-wise multiplication.

##### 2.2.2.2. Bi-directional long- and short-term memory

Bi-directional long- and short-term memory (Bi-LSTM) (23) was capable of capturing long-range sequence dependencies and thus could provide a better context. It processed sequences in both directions before and after and had been shown to yield the best performance when configuring 128 hidden neurons and one layer depth. To prevent overfitting and avoid cooperative adaptation, we set the dropout rate to 0.2 in our predictor. The probability of a 4mC site in the input sequence was represented by one neuron in the output layer, which utilized the sigmoid activation function.


(15) $$\left(\begin{array}{c} i_{t}\\ f_{t}\\ C_{t}{\;}^{\prime } \end{array}\right)=\left(\begin{array}{c} \sigma \\ \sigma \\ \tanh \end{array}\right)\left(\left(\begin{array}{c} W_{x}\\ W_{h}\\ W_{C} \end{array}\right)\left[h_{t-1},c_{t-1}\right]+\left(\begin{array}{c} b_{i}\\ b_{f}\\ b_{g} \end{array}\right)\right)$$

The *i*-th equation for encoding DNA sequence is shown below:


(16) $$C_{t}=i_{t}C_{t}^{\prime }+f_{t}C_{t-1}\;{ο}_{t}=\sigma \left(W_{x}h_{t-1}+W_{h}x_{t}+W_{c}C_{t}+b_{ο}\right)h_{t}={ο}_{t}\tanh C_{t}$$

Where $i_{t}$ represented input gate, $f_{t}$ represented forget gate, ${ο}_{t}$ represented output gate, $C_{t}^{\prime }$ was the auxiliary value of the calculation cell memory $C_{t}$, t was the current time, $W_{x}$, $W_{y}$, $W_{c}$ were the corresponding weight coefficient, $b_{ο}$ was constant at time t, and $h_{t}$ represented the output of the LSTM cell. Since Bi-LSTM consists of two opposite-direction LSTM networks, the i-th nucleotide of the DNA sequence was represented using the following encoding:


(17) $$h_{t}=\left[\underset{h_{t}}{\to }⊕\underset{h_{t}}{\leftarrow }\right]$$

#### 2.2.3. Multi-scale feature fusion

The AFF (34) extracted local features according to global channel attention. The local and global features can be integrated, and the context of multiple-scale features can be obtained through point-wise convolution. This can collect more details from lower-level features and reduce the use of parameters. Its lightweight characteristic made it an ideal replacement for the existing feature fusion module. The construction of AFF is shown in Figure 1. The fused features, Z ∈ R^H × W × C^, were calculated by the following equation:


(18) $$\begin{array}{l} Z=M\left(X+Y\right)⊗X+\left(1-M\left(X⊕Y\right)\right)⊗Y\\ \;=\sigma \left(L\left(X⊕Y\right)⊕G\left(X⊕Y\right)\right)⊗X\\ \;+\left(1-\sigma \left(L\left(X⊕Y\right)⊕G\left(X⊕Y\right)\right)\right)⊗Y \end{array}$$

The σ represented the sigmoid function, ⊕ denoted addition operation, ⊗ denoted element multiplication, and X⊕Y represented the combination of feature X and Y. $M\left(X+Y\right)$ represented the weights of fusion for X; the weights of fusion for Y  were denoted as $1-M\left(X⊗Y\right)$. With values ranging from zero to one, the function of this module was to perform a computation that combined the values of X and Y in a weighted manner. The weighting can be considered as a soft selection or weighted averaging process.

#### 2.2.4. Eliminate noise module

The DRSN set unimportant features to zero by inserting a soft threshold with a trainable shrinkage function and made the high-level features more distinguishable. By combining threshold and depth learning, the information related to noise can be removed and high-quality identification features can be obtained. The soft threshold was defined by the following formula:


(19) $$O=\left\{\begin{array}{c} I-t,I>t\\ \;0,-t\leq I\leq t\\ I+t,I<-t \end{array}\right.$$

where I represented the input feature, O represented the output feature, and t denoted the threshold, which was a positive number. Some activation functions set negative features to zero, such as Rectified Linear Unit (ReLU). Nevertheless, the soft threshold had the ability to assign features that were nearly zero to zero, allowing the network to retain useful negative features. The results of the derivative for output to input had two values, either one or zero, which aided in avoiding the gradient vanishing or explosion. We obtained the derivative formula for formula (19):


(20) $$\frac{\partial O}{\partial I}=\left\{\begin{array}{c} 1,I>t\\ 0,-t\leq I\leq t\\ 1,I<-t \end{array}\right.$$

##### 2.2.4.1. Residual Shrinkage Building Unit with Channel-Wise thresholds

Residual Shrinkage Building Unit with Channel-Wise thresholds (RSBU-CW) was the submodel of DRSN-CW. In this predictor, we used DRSN-CW consisting of two RSBU-CWs to remove noise associated with features. Figure 1 illustrates the architecture of RSBU-CW. The features underwent two one-dimensional convolutions before being transformed into 1D vectors by applying the absolute function and GAP layer, followed by feeding the features into a two-layer FC network. The second layer had multiple neurons equal to the number of channels in input features. After passing the two-layer FC network, the scaling parameter was adjusted to fall within the range (zero and one) by applying the sigmoid function. The output of the FC network was then scaled using the following expression to fall within the range of (zero and one).


(21) $$\alpha_{c}=\frac{1}{1+e^{-z_{c}}}$$

The variable $z_{c}$ referred to the feature of the c-th neuron and was also the output of the two-layer FC network, and the c-th neuron’s scaling parameter was represented by $\alpha_{c}$. For our predictor, the value of C was 256. Following that, the thresholds were calculated by the following equation:


(22) $$\tau_{c}=\alpha_{c}\;\cdot \;\;a\text{verage}\left|x_{i,j,c}\right|$$

We adopted two stacked RSBU-CWs to eliminate the noise-related information in our predictor. The RSBU-CW layer with a soft threshold as shrinkage functions, and was observed through a variety of nonlinear transformation. After a series of experiments, we found that the two stacked RSBU-CWs yielded the best effect in our predictor. As part of the implementation process, we computed the scaling parameter C and corresponding thresholds $\tau_{c}$ for the C-th channel of the features in the features map. i denoted width, j denoted height, and C denoted channel. A value of 256 was chosen for C, and the thresholds were selected to be positive and within an appropriate range to prevent the features of zero-valued output.

#### 2.2.5. Prediction module

In the prediction module, we integrated the feature vectors using a flatten function generated by the dropout layer; the vector was subsequently passed through a fully connected dense layer, which was referred to as dense(n) and contained 32 neurons. In the FC layer, we used the Exponential Linear Units activation function and sigmoid function to produce the ultimate outcomes. The values were scaled to a range of zero to one using the sigmoid function, which represented the probability that was 4mC or non-4mC sites.

The predictor used the Adam optimizer (37) to train, which was efficient, required small memory, and was suitable for large parameter problems. For the binary classification task, binary cross-entropy (12) was utilized to measure the discrepancy between the predicted and target results.

### 2.3. Performance evaluation metrics

To estimate DRSN4mCPred’s effectiveness, we used a range of metrics, including accuracy (ACC), Matthews correlation coefficient (MCC), sensitivity (Sn), specificity (Sp), precision, and $F_{1}-$ score (38), as well as the receiver operating characteristics curve (ROC) and the associated area under the curve (AUC).


(23) $$\text{ACC}=\frac{\text{TP}+\text{TN}}{\text{TP}+\text{FN}+\text{FP}+\text{TN}}$$

(24) $$\text{Sn}=\frac{\text{TP}}{\text{TP}+\text{FN}}$$

(25) $$\text{Sp}=\frac{\text{TN}}{\text{TN}+\text{FP}}$$

(26) $$\text{Precision}=\frac{\text{TP}}{\text{TP}+\text{FP}}$$

(27) $$F_{1}-\text{score}=\frac{2TP}{2\times TP+FP+FN}$$

The abbreviations TP, TN, FP, and FN were used to denote true positive, true negative, false positive, and false negative, respectively. TP represented a total of 4mC sites that were correctly classified into 4mC sites; TN denoted the number of correctly classified non-4mC sites, while FP represented the total number of 4mC sites that were wrongly classified as 4mC. Similarly, FN indicated the total number of non-4mC sites that were wrongly classified as 4mC sites. The performance of DRSN4mCPred was also evaluated using AUC, and its ability to correctly classify 4mC and non-4mC sites was measured.

## 3. Results and discussion

DRSN4mCPred was developed using Keras 2.9.0 and TensorFlow 1.12.0 in Python 3.9. The model was trained using 10-fold cross-validation, where each fold was trained for 50 epochs with a batch size of 142.

### 3.1. Analysis of DNA sequences

**Figure 2:**
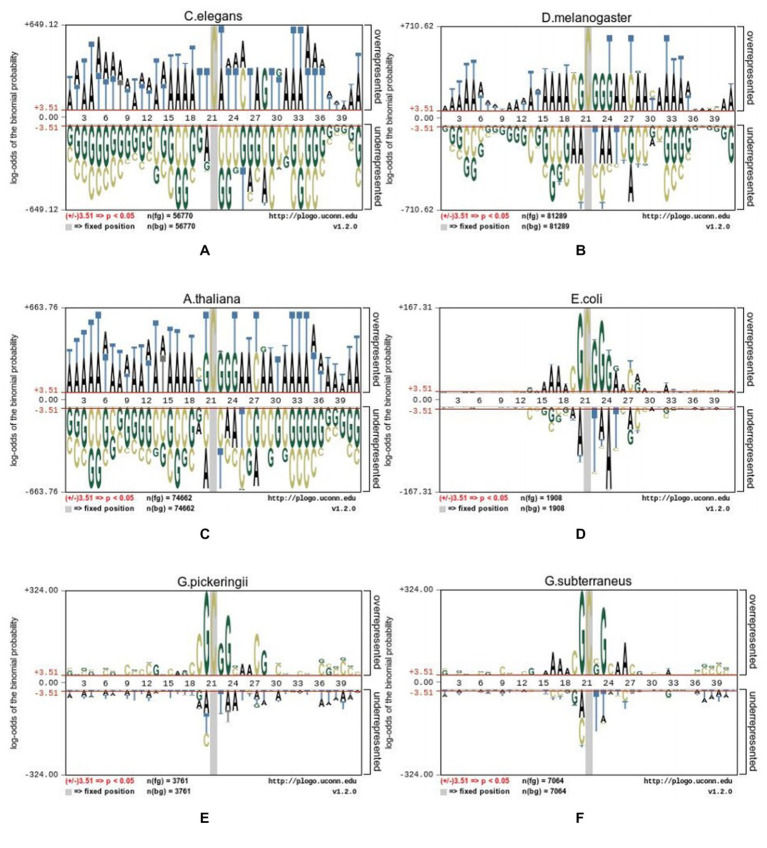
Sequence logo representations of the nucleotide 4mC sites and non-4mC sites on datasets from six species. **(A)** C.elegans, **(B)** D.melanogaster, **(C)** A.thaliana, **(D)** E.coli, **(E)** G.pickeringii, and **(F)** G.subterraneus.

To uncover distribution variations for 4mC and non-4mC sites, the pLogo web server (23) with FLOW (v1.12.0) was used to uncover the differences. Sequence logos were generated to display the nucleotides that were over- or under-represented, indicating the excess and insufficient (*p* = 0.05) at every position of DNA sequences. The Hyb_2021 dataset is displayed in Figure 2 using the pLogo tool, the red horizontal lines indicating a distinct threshold with 3.51 (*p* < 0.05).

The nucleotide distribution of various species was different from each other. For example, in *C. elegans*, adenine (A) was significantly enriched at positions p1-18, 22–25, 27, and 31–41 (*p* < 0.05), while thymine (T) was significantly depleted at positions 20, 26, and 28. In *A. thaliana*, G nucleotides were significantly enriched at positions p22, p23, and p24, while the nucleotides of A were significantly enriched at positions p1-18, 25, 26, 28, 29, and 31–41. In *D. melanogaster*, G was enriched at positions 9, 12, 18, and 20. In *E. coli*, the nucleotides of A were obviously abundant at positions 16, 17, 18, 25, and 28, while G, C, and T were abundant at other positions. In the species of *G. pickeringii* and *G. subterraneus*, the nucleotides were obviously abundant in most upstream and downstream positions, while the nucleotides of T were abundant at position p22.

The above results showed that the position of nucleotides in DNA sequences was a key feature to distinguish the position of 4mC and non-4mC sites. Relying solely on observed features for judgment can result in numerous false positives, so it was necessary to elucidate the related information for the specific location; hence, the method based on machine learning was also necessary, which had been proven to be effective in many fields (Table 1).

**Table 1:** Statistical summary of six species datasets.

Species datasets	Number of train samples		Number of test samples	
	Positive	Negative	Positive	Negative
*D. melanogaster*	81289	81289	28000	28000
*C. elegans*	56770	56770	12147	12147
*A. thaliana*	74662	74661	50966	50966
*E. coli*	1908	1908	160	160
*G. subterraneus*	7064	7064	7813	7813
*G. pickeringii*	3761	3761	1926	1926

### 3.2. Performance on datasets

The datasets of Hyb_2021 and Li_2020 were used for performing performance evaluation tests, respectively. The results of the evaluation tests showed that DRSN4mCPred was an effective predictor for distinguishing 4mC and non-4mC sites. When using DRSN4mCPred to test the Hyb_2021 dataset, we obtained individual AUC values of 0.992, 0.985 0.823, 0.994, 0.992, and 0.992 for the species *C. elegans*, *D. melanogaster*, *A. thaliana*, *E. coli*, *G. subterraneus*, and *G. pickeringii*, as shown in Table 2. Additionally, when we tested DRSN4mCPred on the Li_2020 dataset, the resulting individual AUC values for these species were 0.994, 0.989, 0.946, 0.992, 0.927, and 0.934, separately; the DRSN4mCPred’s AUC average value was 0.964. The above results showed the effectiveness of DRSN4mCPred in distinguishing 4mC sites (Tables 3, 4).

**Table 2:** The individual AUC values for the six species.

Species	AUC	
	Hyb_2021	Li_2020
*C. elegans*	0.996	0.994
*D. melanogaster*	0.990	0.989
*A. thaliana*	0.829	0.946
*E. coli*	0.993	0.992
*G. subterraneus*	0.992	0.985
*G. pickeringii*	0.992	0.986

**Table 3:** Performance on Hyb_2021 datasets.

Species	ACC	Sn	Sp	Precision	F1_score	Auc
*C. elegans*	0.970	0.968	0.972	0.974	0.974	0.996
*D. melanogaster*	0.955	0.961	0.949	0.950	0.955	0.990
*A. thaliana*	0.736	0.889	0.584	0.825	0.841	0.829
*E. coil*	0.978	0.975	0.981	0.981	0.978	0.993
*G. subterraneus*	0.961	0.971	0.952	0.975	0.967	0.992
*G. pickeringii*	0.965	0.947	0.982	0.976	0.973	0.992

**Table 4:** Performance on Li_2020 datasets.

Species	ACC	Sn	Sp	Precision	F1_score	AUC
*C. elegans*	0.969	0.981	0.958	0.959	0.970	0.994
*D. melanogaster*	0.958	0.977	0.938	0.940	0.958	0.989
*A. thaliana*	0.884	0.880	0.889	0.881	0.884	0.946
*E. coil*	0.973	0.976	0.969	0.969	0.973	0.992
*G. subterraneus*	0.952	0.934	0.970	0.969	0.951	0.985
*G. pickeringii*	0.962	0.957	0.966	0.966	0.962	0.986

### 3.3. Analysis of cross-species validation

**Figure 3:**
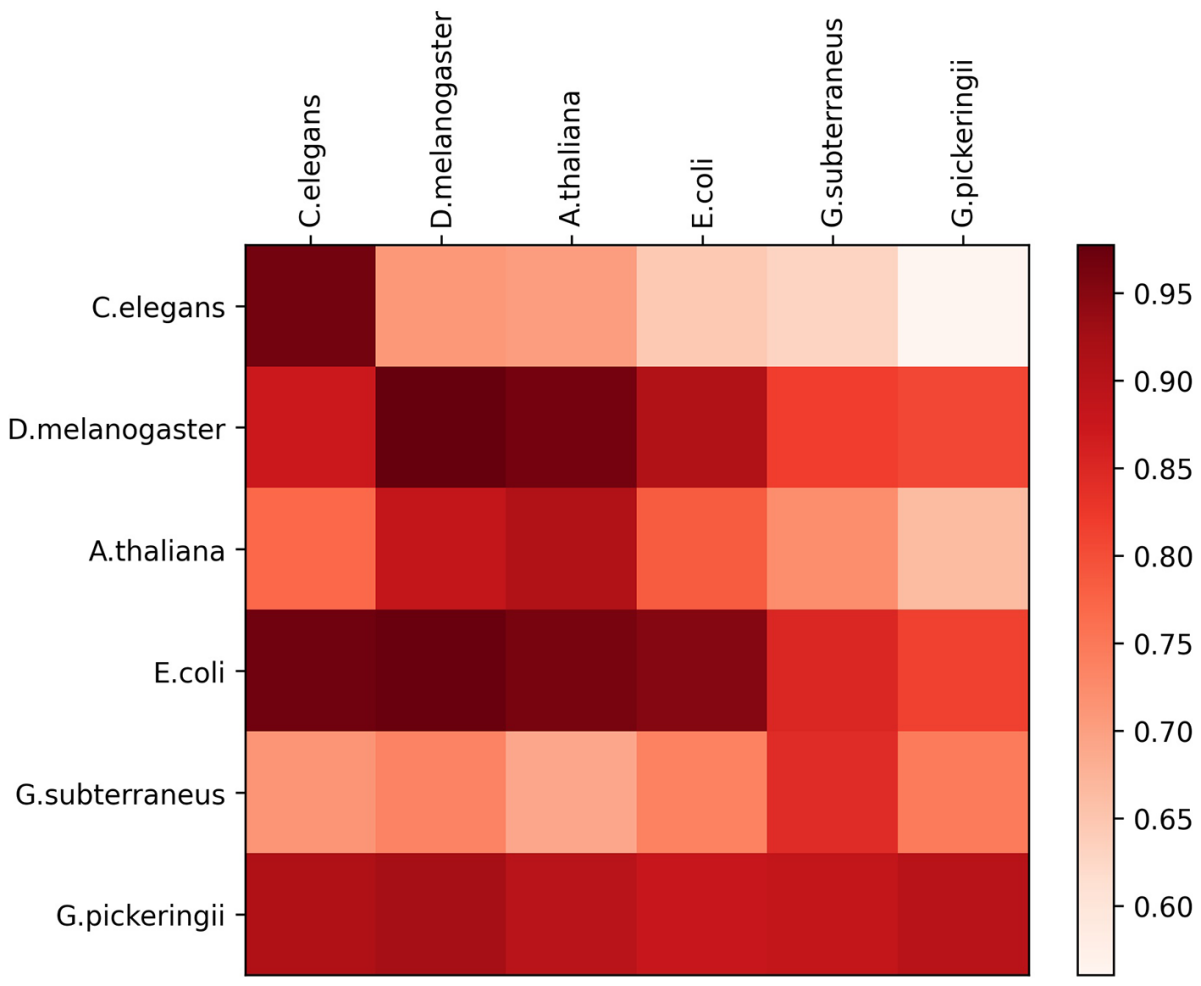
Heat map showing cross-species prediction performance.

The six species benchmark datasets used for cross-species experimental validation were all sourced from the Hyb_2021 dataset. Each DNA sequence has 41 base pairs in length. While one species dataset was used for training the predictive model, the other five species datasets were employed to test the model’s performance. Figure 3 shows the experimental results of the six cross-species using a heat map. The prediction models exhibit a significant performance variation among the six species. The accuracy of the predictive models was the lowest for *E. coli* species when applied to the species of *C. elegans*, *D. melanogaster*, and *A. thaliana*. However, the models based on datasets of *C. elegans* species, *D. melanogaster* species, and *A. thaliana* species demonstrated excellent accuracy when predicting each other, with 96.69, 97.73, and 90.97% accuracy, respectively. The models based on the species of *G. subterraneus* and *G. pickeringii* also achieve high accuracy in predicting the four species.

Overall, the prediction model based on the features of extraction by DRSN4mCPred was highly effective in identifying DNA 4mC sites across species, demonstrating the strong categorical information available in the extracted features. This suggests that our DRSN4mCPred predictive model can effectively extract significant information from DNA sequences for identifying both 4mC and non-4mC sites of DNA.

## 4. Conclusion

This paper introduced the DRSN4mCPred model to discriminate DNA 4mC sites. To verify its accuracy, we used two datasets Hyb_2021 and Li_2020, which comprise species such as *C. elegans*, *D. melanogaster*, *A. thaliana*, *E. coli*, *G. subterraneus*, and *G. pickeringii*. On the Hyb_2021 dataset, the AUC of DRSN4mCPred for the species of *C. elegans*, *D. melanogaster*, *E. coli*, and *G. pickeringii* achieved 0.996, 0.995, 0.995, 0.991, and 0.992. Additionally, DRSN4mCPred’s performance on the Li_2020 database was also very good.

Incorporating DSBU-CW into the model of DRSN4mCPred resulted in improved prediction performance, which effectively eliminated noise-related features and captured critical features. The use of multi-scale channel attention and attentional feature fusion to automatically learn both high-level and low-level features leads to better accuracy in distinguishing 4mC sites and non-4mC sites. This research could offer assistance to the diagnosis and treatment of gastrointestinal cancer in the precision medicine era.

## Data availability statement

The datasets presented in this study can be found in online repositories. The names of the repository/repositories and accession number(s) can be found at: https://github.com/YingLiangjxau/Hyb4mC/tree/main/Hyb4mC.

## Author contributions

YC contributed to the conception of the study. XY performed the experiment, contributed significantly to the analysis, and wrote the manuscript. JR, RZ, and HL helped perform the analysis with constructive discussions. All authors contributed to the manuscript revision, read, and approved the submitted version.

## Funding

This research was funded by the National Natural Science Foundation of China (Nos. 62262016 and 62262019), the Hainan Provincial Natural Science Foundation Innovation Research Team Project (620CXTD434), the High-level Talent Project of Hainan Provincial Natural Science Foundation (620RC557), the program of the Scientific Research Foundation of Hainan University [KYQD(ZR)1859], the Hainan Provincial key research and development plan of China (No. ZDYF2021GXJS200), and the Hainan Provincial Natural Science Foundation of China (No. 621QN241).

## Conflict of interest

The authors declare that the research was conducted in the absence of any commercial or financial relationships that could be construed as a potential conflict of interest.

## Publisher’s note

All claims expressed in this article are solely those of the authors and do not necessarily represent those of their affiliated organizations, or those of the publisher, the editors and the reviewers. Any product that may be evaluated in this article, or claim that may be made by its manufacturer, is not guaranteed or endorsed by the publisher.
